# A Boy with Rash and Joint Pain Diagnosed with Scurvy: A Case Report

**DOI:** 10.21980/J89H1X

**Published:** 2021-10-15

**Authors:** James Tran, Yih Ying (Eva) Yuan

**Affiliations:** * Morehouse School of Medicine, Atlanta, GA; ^ Emergency Medicine Specialists of Orange County (EMSOC), Children’s Hospital of Orange County, Division of Pediatric Emergency Medicine, Orange, CA

## Abstract

While rare, scurvy is a disease the emergency physician should continue to consider in the differential of bruising and joint pain. Although these symptoms are nonspecific, the astute physician should remain open-minded even when scurvy is not likely to be seen in an economically stable country. Contrary to this, we report a case of a young boy in the United States with scurvy who presented with a rash and limp. The diagnosis of scurvy can be made alone by a thorough history, but radiologic studies and pertinent labs also helped. Early diagnosis resulted in adequate vitamin supplementation and quick recovery of symptoms thereby avoiding unnecessary testing and lowering the cost of care.

**Topics:**

Scurvy, pediatric, vitamin C deficiency, nutritional deficiencies.

**Figure:**
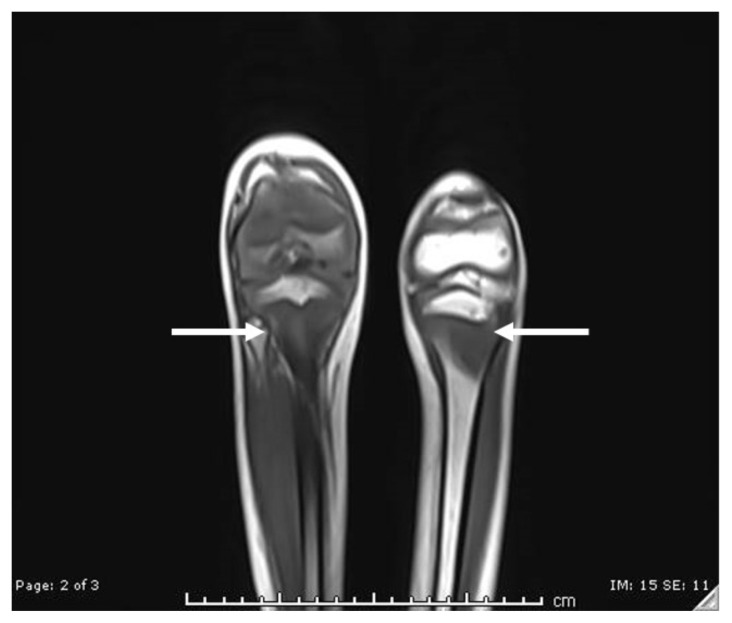


**Figure:**
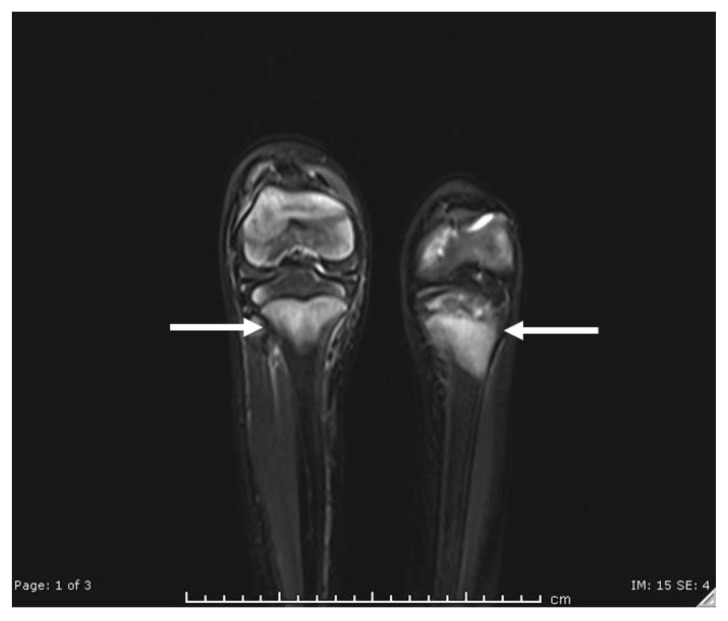


## Brief introduction

Scurvy results from vitamin C deficiency and is characterized by increased bleeding risk, given its role in collagen formation and wound healing. As with other nutritional deficiencies, scurvy is seen in populations lacking a balanced diet, namely developing countries, the elderly, and those with eating disorders. In the United States, where food is more abundant and fortified with essential vitamins and minerals, scurvy is less likely to be expected. Nonetheless, scurvy can still be seen in select groups. We report a case of a young boy in the United States with scurvy who presented with a rash and limp.

## Presenting concerns and clinical findings

A 7-year-old male with autism presented to the emergency department with one month of progression of symptoms, including rash and bilateral knee pain, now with an inability to ambulate. The rash started on his lower extremities two weeks prior and now spread to his hands. He also had bleeding gums and increasing epistaxis over the past month. He was first seen at an outside hospital a week prior, where labs and X-rays of his knees were unremarkable except for slight anemia. He was diagnosed with Henoch-Schoenlein Purpura (HSP) and started on a 5-day course of prednisone. His family history included relatives with rheumatoid arthritis, Raynaud's disease, and anemia.

On presentation to the ED, the patient's vitals were heart rate 87 beats/min, blood pressure 116/65 mm Hg, respiratory rate 22 breaths/min, oxygen saturation 100% on room air, and temperature 36.7 °C. He appeared pale and tired. His skin exam showed multiple bruises on thighs and hands, and petechiae on bilaterally lower extremities. There was tenderness to palpation of bilateral knees with limited ROM, but no swelling or erythema. The oropharyngeal exam showed swollen gums. The rest of the exam was normal. Labs in the ED, including complete blood count (CBC), comprehensive metabolic panel (CMP), C reactive protein (CRP), erythrocyte sedimentation rate (ESR), disseminated intravascular coagulation (DIC) panel, ferritin, lactate dehydrogenase (LDH), reticulocyte count, uric acid, and urinalysis (UA) were only remarkable for microcytic anemia (hemoglobin 10.8 gm/dL and mean corpuscular volume 74), elevated ESR of 49 mm/hr, and elevated LDH of 970 units/L. He was admitted for further evaluation. Differential diagnosis included HSP, COVID vasculitis, juvenile idiopathic arthritis, bleeding disorder, and leukemia/lymphoma.

## Significant findings

His lower extremity magnetic resonance imaging (MRI) findings showed abnormal signals in his knees, which were most consistent with scurvy. The white arrows on the T1-weight sequence indicate hypointensity (decreased signal or darker region) of the knees. The white arrows in the T2-weighted short-tau inversion recovery (STIR) sequence indicate hyperintensity (increased signal or brighter region) in an MRI of the knees.

## Patient course

After an extensive negative rheumatologic and hematologic workup as an inpatient, further history revealed the patient had a severely limited diet (only eating crackers, chips, and grilled cheese). Subsequent laboratory workup showed vitamin D 25-hydroxy D level <6.0 Ng/mL (<10 Ng/mL indicating severe deficiency), and iron deficiency anemia (iron level 31 mcg/dL, transferrin saturation 10%). Ascorbic acid was initially drawn but due to lab error was not resulted by the time the patient had completed vitamin supplementation. Copper, folate, zinc, and selenium levels were all within normal limits.

The patient was ultimately diagnosed with scurvy and vitamin D and iron deficiency. He was discharged after receiving multivitamins, cholecalciferol, vitamin C, and help from the nutritionists to establish a balanced diet. During his two-week follow-up appointment, his parents reported improvement in his weight-bearing, energy, rash, bleeding, and skin color.

## Discussion

Scurvy, defined as symptomatic vitamin C deficiency, is a rare disease affecting approximately 6.2% of the United States population greater than 9 years old. Only 1.1% of males and females between 9 to 13 years old are at risk.^1^ Given vitamin C's role in collagen synthesis, patients with scurvy often present with signs of increased bleeding, which can become apparent 1–3 months after insufficient vitamin C intake.^2^ The most specific clinical presentation are follicular hyperkeratosis and perifollicular hemorrhage with corkscrew hairs. Other common signs include gingivitis with bleeding gums and hemorrhagic skin lesions.^3^ Musculoskeletal pain may also be present when weakened blood vessels hemorrhage into the muscles or periosteum, manifesting as generalized ecchymoses or bone pain.^4^ Many of these were seen in our patient.

In addition, a single vitamin deficiency often presents with other concurrent dietary inadequacies, which was also seen in our patient who had vitamin D and iron deficiency.^1^ Vitamin D deficiency likely further worsened his knee pain, and anemia contributed to his decreased energy and pallor. Because vitamin C also acts as a co-factor to increase the absorption of nonheme iron in the gut, it is not surprising to see a patient with vitamin C deficiency also having iron deficiency.^5^

Given the nonspecific nature of the symptoms, the diagnosis of scurvy can be clouded by more common diagnoses such as oncologic (leukemia, lymphoma), hematologic (bleeding disorders), or rheumatologic (vasculitis, arthritis). Hence the workup of our patient (and many similar patients in the literature) included exploring these possible etiologies, particularly neoplastic causes, given its pressing nature.^6^ In our case, a more thorough history and a higher suspicion for nutritional deficiencies could have resulted in the earlier diagnosis of scurvy.

The diagnosis of scurvy is made clinically rather than by measuring vitamin C or ascorbic acid levels. To correlate the symptoms, plasma ascorbic acid concentration level can be measured once vitamin C deficiency is suspected with features occurring at a serum concentration less than 0.2 mg/dL (11.4 micromol/L).^7^ While also not necessary for making the diagnosis, imaging studies in patients with scurvy are often obtained to evaluate their musculoskeletal pain. The MRI finding in our patient is consistent with what has been described in the literature: "increased T2-W signal in the marrow cavities of the metaphyses, particularly of the bones adjacent to the knee."^8^ Similarly, bone marrow aspiration or biopsy is unnecessary, but it has been reported as part of the workup in several pediatric patients prior to the eventual diagnosis of scurvy.^9^

The best confirmation for the diagnosis of scurvy lies in the improvement of symptoms following appropriate ascorbic acid treatment. The dose and duration are patient-specific, but treatment for children is usually 100 to 300 mg of vitamin C daily for one month or until full recovery.^2^ Symptoms can improve within days to weeks, as was seen with our patient.

Scurvy is a rare disease resulting from vitamin C deficiency.^1^ As with many nutritional deficiencies, it is more prone in low resource populations, the elderly, and those with eating disorders. Although patients with autism do not precisely fit into this last category, their restrictive behaviors can be severe enough to cause inadequate nutrient intake. However, several studies done exploring the nutritional intake in children with autism have yielded mixed findings, some reporting inadequate intake while others reporting no difference when compared to children without autism.^10^ Nonetheless, suspecting a nutritional deficiency in children with autism presenting with bleeding and a limp can result in earlier diagnosis and lower cost of care.

## Supplementary Information




