# Supplementary figures and images for: A Boy with Rash and Joint Pain Diagnosed with Scurvy: A Case Report
Source: J Educ Teach Emerg Med. 2021 Oct 15;6(4):V6–8. doi: 10.21980/J89H1X (PMC10332736; doi:10.21980/J89H1X)

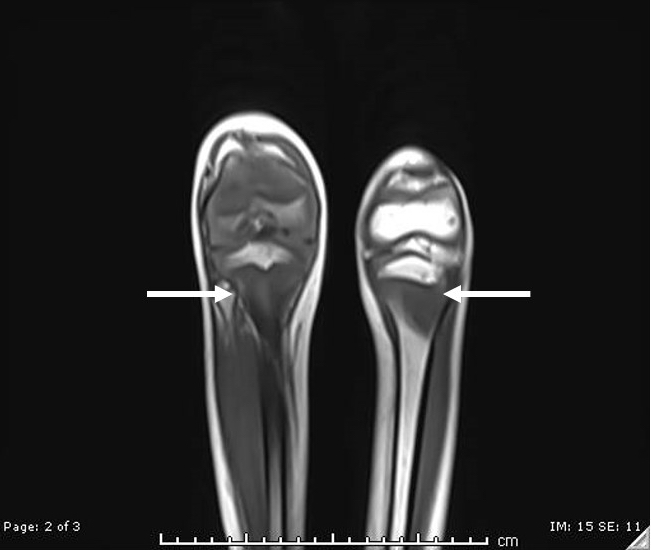

Supplement: Supplementary file 1 [file JETem-6-4-V6-supp1.jpg]

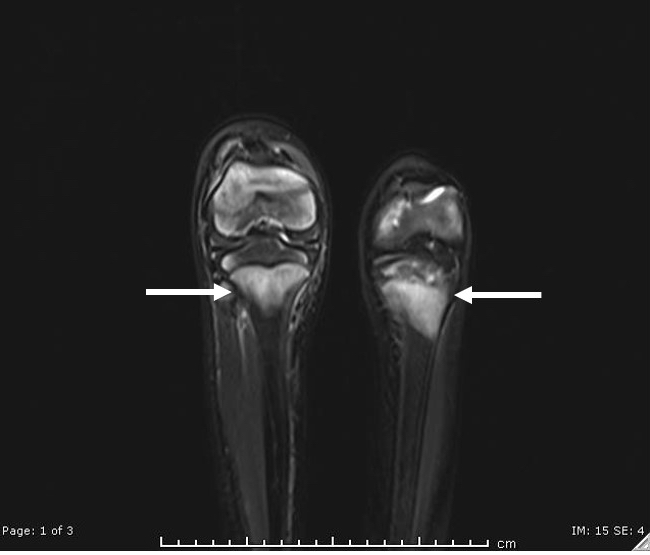

Supplement: Supplementary file 2 [file JETem-6-4-V6-supp2.jpg]
